# Creating a regular array of metal-complexing molecules on an insulator surface at room temperature

**DOI:** 10.1038/s41467-020-20189-x

**Published:** 2020-12-21

**Authors:** Simon Aeschlimann, Sebastian V. Bauer, Maximilian Vogtland, Benjamin Stadtmüller, Martin Aeschlimann, Andrea Floris, Ralf Bechstein, Angelika Kühnle

**Affiliations:** 1 Institute of Physical Chemistry, Johannes Gutenberg University Mainz, Duesbergweg 10-14, 55099 Mainz, Germany; 2 Graduate School Materials Science in Mainz, Staudingerweg 9, 55128 Mainz, Germany; 3 Physical Chemistry I, Department of Chemistry, Bielefeld University, Universitätsstraße 25, 33615 Bielefeld, Germany; 4 Department of Physics and Research Center OPTIMAS, University of Kaiserslautern, Erwin-Schrödinger-Straße 46, 67663 Kaiserslautern, Germany; 5 School of Chemistry, University of Lincoln, Brayford Pool, Lincoln, LN67 PS UK

**Keywords:** Scanning probe microscopy, Surface assembly, Nanoscale materials

## Abstract

Controlling self-assembled nanostructures on bulk insulators at room temperature is crucial towards the fabrication of future molecular devices, e.g., in the field of nanoelectronics, catalysis and sensor applications. However, at temperatures realistic for operation anchoring individual molecules on electrically insulating support surfaces remains a big challenge. Here, we present the formation of an ordered array of single anchored molecules, dimolybdenum tetraacetate, on the (10.4) plane of calcite (CaCO_3_). Based on our combined study of atomic force microscopy measurements and density functional theory calculations, we show that the molecules neither diffuse nor rotate at room temperature. The strong anchoring is explained by electrostatic interaction of an ideally size-matched molecule. Especially at high coverage, a hard-sphere repulsion of the molecules and the confinement at the calcite surface drives the molecules to form locally ordered arrays, which is conceptually different from attractive linkers as used in metal-organic frameworks. Our work demonstrates that tailoring the molecule-surface interaction opens up the possibility for anchoring individual metal-complexing molecules into ordered arrays.

## Abstract (web summary)

Arrays of ordered metal atoms on bulk insulating materials are promising for future applications, such as optoelectronics and data storage. Here, the authors demonstrate a strategy to create an ordered metal array based on tailored anchoring and hard-sphere repulsion of metal-complexing molecules.

## Introduction

Molecules on surfaces^1,2^ offer a great variability of creating functional structures for future technologies^3–5^, including molecular electronics^6,7^, data storage^8,9^, sensing^10^, and catalysis^11,12^. This is particularly true when considering molecules that carry or coordinate metal atoms, e.g., in structures formed from metalated molecules such as metaloporphyrins^13^ and metalocyanines^14^ as well as surface-supported metal–organic frameworks^15,16^ or metal–organic coordination networks^17^. All these structures offer additional interesting magnetic and superior catalytic^11^ activities^15,18–20^. Creating functional molecular structures on surfaces is based on the capability to anchor molecules to specific adsorption sites under operation condition, i.e., ideally at room temperature. For many applications, in particular molecular electronics, data storage, sensing, and catalysis, it is highly desirable to decouple the electronic structure of the molecules—and especially coordinated metal atoms—from the underlying substrate surface^21^, which can be achieved by using a bulk insulator as substrate. Many insulator surfaces, however, are rather inert^22,23^, which is why organic molecules often exhibit weak binding and high diffusivity on bulk insulator surfaces. In recent years, several strategies have been developed for anchoring organic molecules to insulator surfaces, including molecule functionalization for specific binding or increasing electrostatic interactions^24–30^. However, stabilizing single molecules on an electrically insulating support sample at room temperature remains a great challenge, and examples are limited to clusters^31^ rather than single molecules. As stabilizing single molecules on bulk insulators bear great potential for future applications, it is highly desired to develop strategies to prevent the molecules from clustering, but still exhibiting a sufficient molecule–substrate interaction for anchoring. So far, it has been demonstrated that single molecules can be trapped at defects on rutile titanium dioxide (110)^32^. However, in present of the defects, the band gap of TiO_2_ is strongly reduced, and hence, the insulating properties vanish^33^. As far as we know, cytosine trimers on the calcium fluoride (111) cleavage plain have been identified to be the smallest observed stable configuration at room temperature on a bulk insulator^31^. For cytosine monomers, a diffusion barrier of 0.5 eV has been determined^31^, which corresponds to a high mobility at room temperature. Until now, only larger monomers, such as 4,4′-di(4-carboxyphenyl)-6,6′-dimethyl-2,2′-bipyridine on NiO (001), are found to have a reduced mobility on insulators^34^. But in the latter case, due to a strong molecule–molecule interaction, cluster or even larger molecular islands are formed at increased coverage. Thus, to the best of our knowledge, at room temperature no immobile and stable monomers on bulk insulators have been observed so far.

**Fig. 1:**
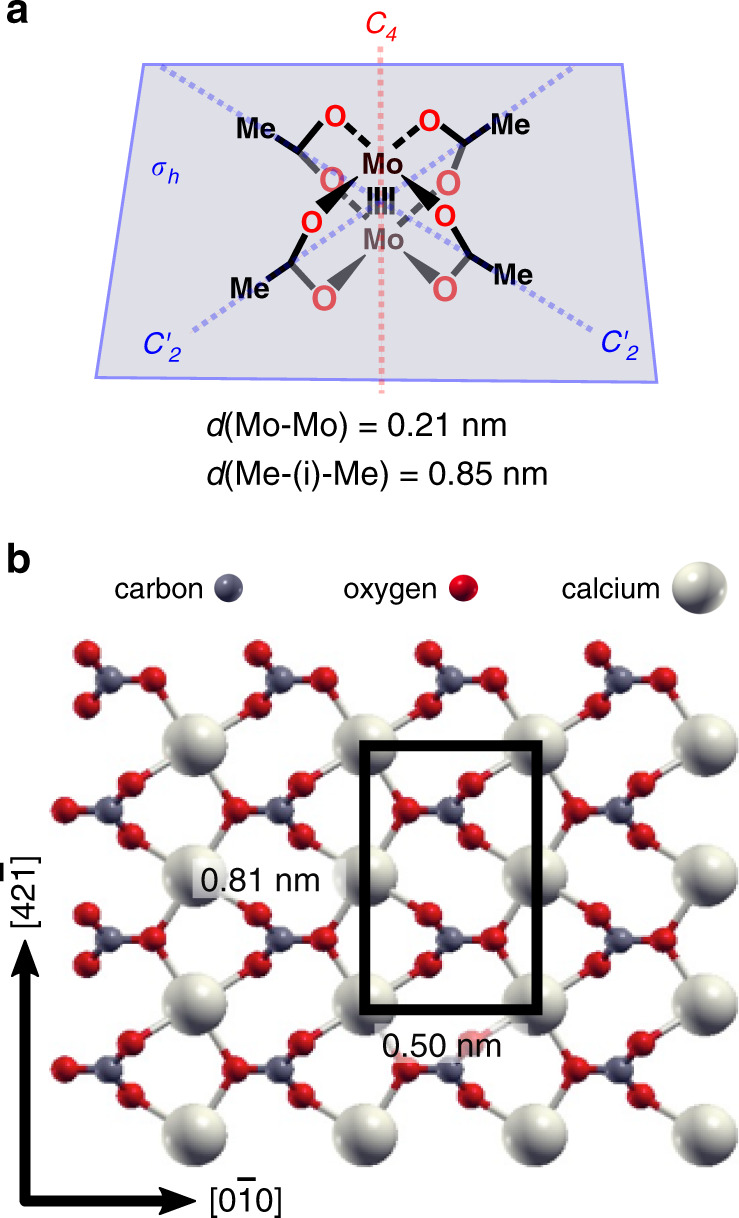
Model of the system studied here. **a** The MoMo molecule adsorbed on **b** the calcite (10.4) surface. The surface unit cell is marked with a black rectangle.

Here, we present the formation of an ordered array of individual dimolybdenum tetraacetate (Mo_2_(O_2_CMe)_4_, in this work referred to as MoMo, Fig. 1a) molecules on the natural (10.4) cleavage plane of the bulk insulator calcite, the most stable modification of calcium carbonate (for a surface structure see Fig. 1b). Based on our atomic force microscopy (AFM) images carried out in the frequency modulation mode under ultrahigh vacuum conditions, we provide experimental evidence of the fact that the MoMo molecules neither diffuse nor rotate on the surface held at room temperature. Our results indicate that the molecules adopt a specific adsorption position that is governed by a perfect match between the charge distribution within the molecule and at the surface. The resulting electrostatic interaction leads to a strong anchoring of single molecules in a well-defined, arrested geometry with apparently high diffusion and rotation barriers.

When increasing the molecule coverage on the surface, the molecules arrange in an ordered array. As MoMo contains metal atoms, our work constitutes an example for creating an ordered array of metal atoms on a bulk insulator material. Thus, we arrive at a structure similar to the situation in surface-supported metal–organic frameworks. Interestingly, the observed order is not induced by intermolecular attraction, but a strong, side-specific surface interaction and a hard-sphere repulsion between the molecules. Thus, in contrast to the attractive linkers employed in surface-supported metal–organic frameworks, in our system the order is based on a combination of intermolecular repulsion and substrate specific adsorption positions. Therefore, this work describes a prime example of single, metal-coordinating molecules strongly anchored onto an insulator surface at room temperature and, hence, elucidates an alternative route for creating an ordered metal array.

## Results

### Molecule adsorption position and geometry

**Fig. 2:**
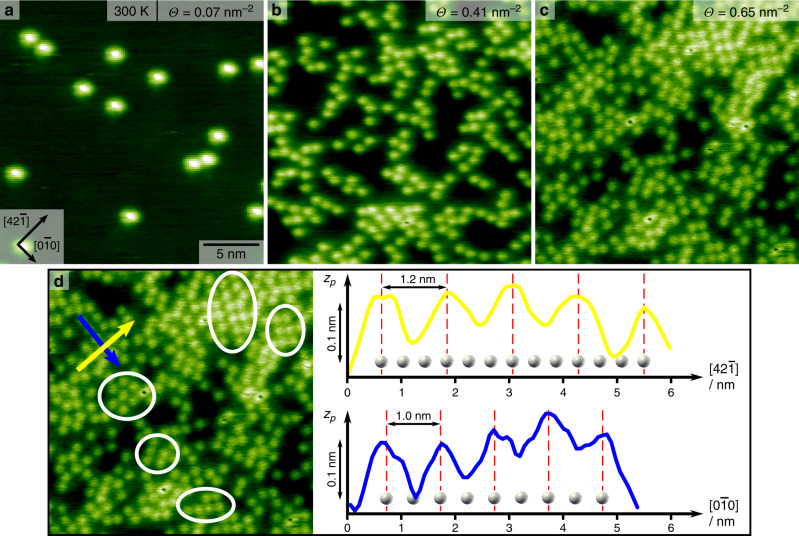
AFM image of MoMo molecules on calcite (10.4) as a function of coverage. **a** At a low coverage of 0.07 molecules per nm^2^, single molecules are obtained. **b** Upon increasing the coverage to 0.41 molecules per nm^2^, areas with higher molecule density are seen, however no ordered inner structure is visible. **c** At a high coverage of 0.65 molecules per nm^2^, partly ordered domains can be observed. **d** Some ordered domains are marked with a white circle. Height profiles along the $\left[ {42\bar 1} \right]$ (yellow arrow) and $\left[ {0\bar 10} \right]$ (blue arrow) direction are displayed. The white calcium ions indicate the substrate lattice. MoMo adsorbs exclusively on identical adsorption sites.

Upon submonolayer deposition of MoMo onto calcite (10.4) held at room temperature, individual features can be recognized in AFM images as shown in Fig. 2. From the size of these bright features and the high-resolution images presented below, we can clearly assign the bright features to single molecules. In Fig. 2a, a representative image is given after low-coverage deposition (~0.07 molecules per nm^2^). Here, a calcite terrace is seen with individual molecules that are randomly scattered over the surface. Each molecule is imaged as a protrusion with radial symmetry. No inner structure can be recognized in this image. When increasing the coverage to ~0.41 molecules per nm^2^, areas with higher molecule density can be seen as in Fig. 2b. At this coverage, no obvious order can be observed in the areas with higher density. The latter finding is changed when further increasing the coverage to 0.65 molecules per nm^2^ as in Fig. 2c. Now, some short-range order becomes apparent in small domains in the areas with high molecule density (marked with white circles in Fig. 2d), e.g., in the upper right area of the image. In Fig. 2d, height profiles are taken along the main symmetry directions $\left[ {42\bar 1} \right]$ (see yellow arrow) and $\left[ {0\bar 10} \right]$ (see blue arrow) of the calcite substrate. The maxima in these height profiles are commensurate with the periodicity of the underlying substrate, which is indicated by white calcium ions. Consequently, MoMo molecules seem to adopt a favorite adsorption site on the surface.

**Fig. 3:**
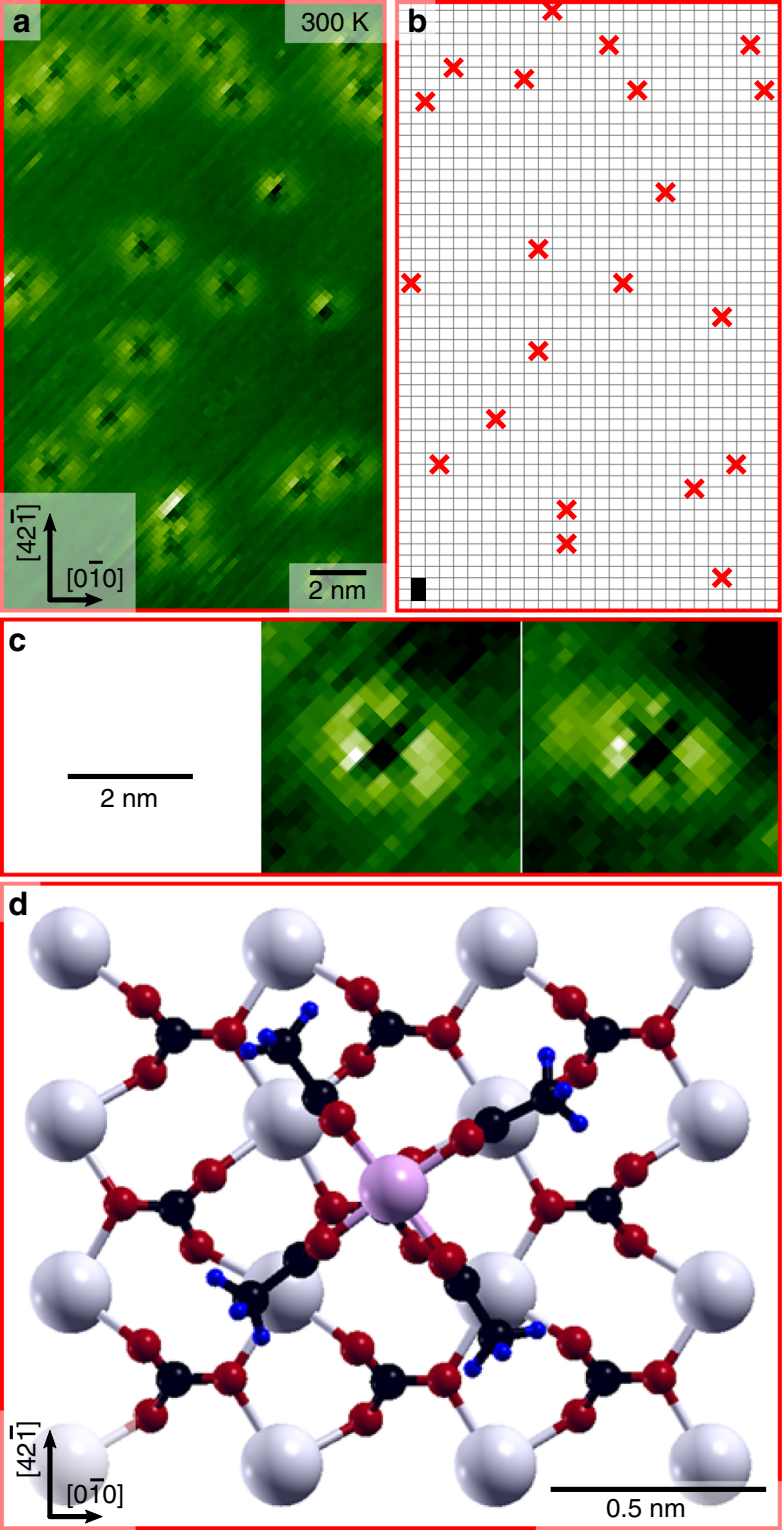
MoMo adsorption geometry on calcite (10.4). **a** High-resolution AFM image of MoMo molecules on calcite (10.4) held at room temperature. The molecules are imaged as dark, cross-like structure surrounded by a bright rim. All crosses are oriented in the same direction. **b** Sketch of the position of the crosses to illustrate the uniform orientation and adsorption site. A calcite (10.4) unit cell is marked with a black rectangle in the bottom left corner. Due to nonlinear thermal drift, a slight deviation between **a** and **b** is visible. **c** Zoom onto two molecules highlighting the cross-like structure. **d** Top view of thermodynamically most stable MoMo adsorption geometry on calcite (10.4) determined by DFT calculations.

To shed light onto the adsorption geometry of the MoMo molecule on calcite (10.4), we next perform high-resolution images with the tip close to the surface (i.e., large excitation frequency shift values) as given in Fig. 3. As can be seen in Fig. 3a, the molecules now appear as features with a bright rim and a dark cross-like structure in the center. The here observed change in contrast of the molecules from bright dots to dark cross-like objects upon decreasing the tip–sample distance can be explained as contrast inversion upon entering into the repulsive regime^35,36^. Interestingly, the appearance of the dark cross in the inner part of the molecule sheds light onto important details about the molecule adsorption geometry. First, the *C*_*4*_ symmetric shape of the inner cross indicates that the molecules lie flat on the surface rather than standing upright. Second, all crosses show the same orientation with respect to the $\left[ {0\bar 10} \right]$ crystal direction as evidenced by the reproduction of the crosses in Fig. 3b. Therefore, we conclude that MoMo cannot rotate freely at its adsorption spot on the calcite surface. Still, a possibility exists for a stepwise rotation of ±90°; however, since MoMo possesses a *C*_*4*_ symmetry this rotation would not change the adsorption configuration. Hence, MoMo is effectively locked in one adsorption configuration on the calcite surface (see zoom images in Fig. 3c). Third, as the molecules are imaged intact in our AFM measurements, jumps from one to the other adsorption site are very rare. The latter finding already provides a first indication for a high diffusion barrier experienced by MoMo on calcite (10.4).

To elucidate the adsorption geometry, we performed density functional theory (DFT) calculations of a single MoMo molecule on calcite (10.4). Figure 3d shows the most favorable adsorption geometry out of 64 tested molecule configurations (see Supplementary Discussion, DFT Calculations of Single MoMo on Calcite (10.4), Supplementary Fig. 1). In order to identify the nature of the strong and site-specific adsorption of MoMo on calcite, we performed an extensive charge analysis (a detailed description of the charge analysis can be found in the Supplementary Discussion, Löwdin Charge Analysis, Charge Density and Charge Displacement Field). Our calculations reveal that the adsorption geometry is predominantly governed by electrostatics: The molecule core composed of the two positively charged molybdenum atoms ($\approx + 1.0\,e$) is centered on top of an overall negatively charged carbonate group ($\approx - 0.8\,e$). Moreover, the molecule aligns in a way that four of the oxygen atoms ($\approx - 0.4\,e$) of the molecule can interact with the positively charged calcium ions ($\approx + 1.0\,e$) of the surface. The formation of a covalent bond between the molecule and substrate or any surface mediated effects like electron screening are basically ruled out (see Supplementary Discussion, Löwdin Charge Analysis, Charge Density and Charge Displacement Field). This adsorption geometry readily explains the experimentally observed cross-like features in our AFM images.

### Diffusion analysis

**Fig. 4:**
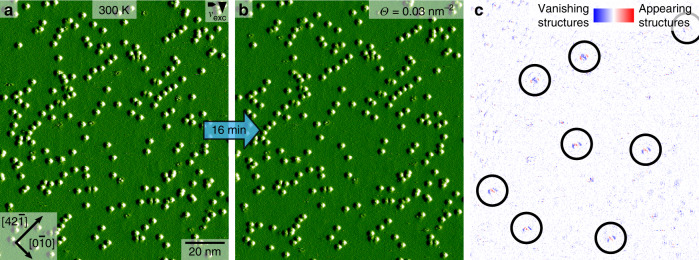
Diffusion study of MoMo on calcite (10.4). **a**, **b** Two consecutive AFM images with a time lapse of 16 min of the same area. **c** Difference image of the images shown in **a** and **b**, illustrating that 8 (marked by black circles) out of 241 molecules change position along the $\left[ {42\bar 1} \right]$ direction. In contrast, no molecules are found to move along the $\left[ {0\bar 10} \right]$ direction, which constitutes a clear sign of anisotropic diffusion. Blue and red colors label disappearing and appearing features, respectively. The sequence of three changes (blue, red, blue or red, blue, red) is a consequence of the fact that the molecules show a depression to the left.

Next, we further elucidate the mobility of the molecules on the surface and the above-mentioned locally ordered domains. When comparing consecutive images taken with a time lapse of 16 min, only few molecules change position along the $\left[ {42\bar 1} \right]$ direction (Fig. 4), corresponding to a diffusion rate of about $\nu _{{\mathrm{diff,}}\,{\mathrm{300K}}} = 3.5 \cdot 10^{ - 5}\,\mathrm{s}^{ - 1}$. This demonstrates the low diffusitivity of the molecules at room temperature. By means of the Arrhenius equation

1 $$\nu _{{\mathrm{diff}}} = \nu _0 \cdot e^{ - \frac{{E_{{\mathrm{diff}}}}}{{k_BT}}},$$

an estimate for the diffusion barrier $E_{{\mathrm{diff}}}$ can be obtained. Assuming an attempt frequency of $\nu _0 = 10^{12}\,{\mathrm{s}}^{ - 1}$ (motivated by the phonon frequency at room temperature) results in a diffusion barrier of $E_{{\mathrm{diff}}} = 1.0\,{\mathrm{eV}}$. In order to gain more insights into the diffusion process, DFT calculations of a molecule moving along calcite’s main axes $\left[ {42\bar 1} \right]$ and $\left[ {0\bar 10} \right]$ were performed (see Supplementary Discussion, Diffusion Energy Barriers Along the $[0\bar 10]$ and $\left[ {42\bar 1} \right]$ Directions Calculated by Nudged Elastic Band (NEB) for more details). Along the $\left[ {0\bar 10} \right]$ direction, a large energy barrier of 1.17 eV prohibits any noticeable molecule diffusion along this direction at room temperature. In contrast, along the $\left[ {42\bar 1} \right]$ direction, a smaller energy barrier of 0.88 eV is found, explaining the anisotropic movement of a few molecules observed in the experiment in Fig. 4. So far, such high diffusion barriers are typically found for molecules adsorbed on metals rather than electrical insulator materials^23^. Upon increasing the temperature to 327 K, the diffusion rate already increases roughly by a factor of ten to $\nu _{{\mathrm{diff,}}\,{\mathrm{327K}}} = 4.0 \cdot 10^{ - 4}\,\mathrm{s}^{ - 1}$ (a detailed description of the diffusion analysis at 327 K can be found in the Supplementary Discussion, Diffusion Analysis at 327 K). Therefore, at elevated temperatures above 400 K, we expect the molecules to overcome the diffusion barrier in a reasonable time frame.

### Annealing analysis

As the molecules cannot diffuse at room temperature, the absence of islands at low coverage might be simply prohibited by the fact that arriving molecules are kinetically trapped at their positions. To investigate whether island formation can be induced when the diffusion barrier can be overcome, we perform annealing experiments. In this experiment, we anneal the sample at a given temperature for 1 h, let it cool down to room temperature, and image the surface. This experiment is repeated with increasing annealing temperature to observe the resulting structural changes in the molecular pattern on the surface (see Supplementary Discussion, Annealing Experiments, Supplementary Fig. 12). Although the molecules start to move at elevated temperatures, the qualitative arrangement in a randomly scattered fashion remains basically unchanged up to a temperature of ~740 K, at which we had difficulties to regain stable imaging conditions as irregular clusters start to form. We tentatively ascribe the latter finding to the fact that the molecules start to decompose and the fragments tend to cluster. We note that MoMo is known to desorb from the metal surface Cu(111) at a temperature below 500 K (personal communication with Lu Lyu, TU Kaiserslautern), indicating the strong anchoring on a bulk insulator that is achieved in this study. For MoMo on calcite (10.4), an adsorption energy of 2.25 eV is obtained in our DFT calculations, which readily explains the high desorption temperature. To summarize, although the molecules start to move at elevated temperatures, they do not assemble to form dense islands. This is an interesting finding as it provides evidence for the fact that there is no detectable attractive interaction between the molecules.

### Formation of a regular molecule array

With this observation, we return to our above finding of local order in small domains when the molecule–surface density is high. Assuming an identical adsorption position for all molecules and a simple hard-sphere repulsion between the molecules, we can draw unit cells for arrangements in which the molecules are densely packed, yet assuming no attractive interaction between the spheres.

**Fig. 5:**
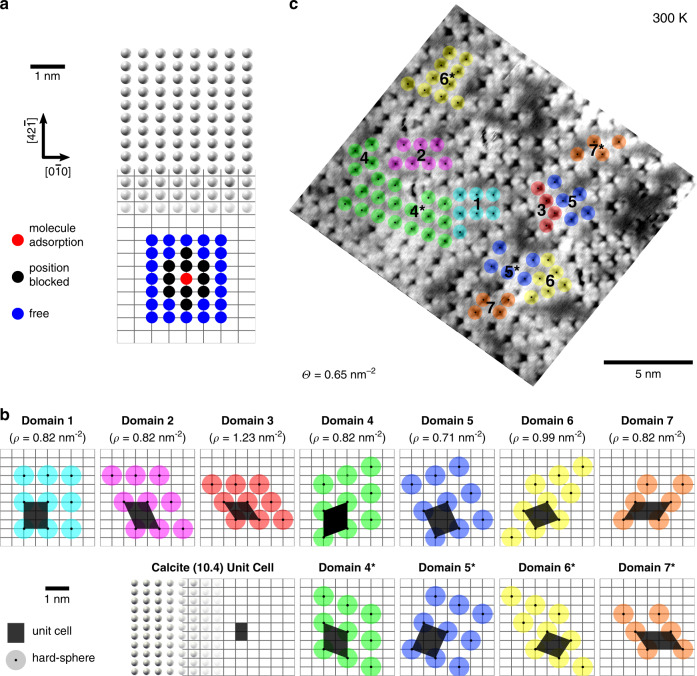
Ordered molecular arrays at high coverage. **a** Spheres representing carbon atoms and the gray grid illustrate the periodicity of the calcite (10.4) surface. Each crossing point of the grid indicates a possible MoMo center adsorption position. A molecule centered at the red dot blocks direct neighbor adsorption positions (marked with black dots) for other molecules. Blue dots indicate nearby free adsorption positions. A diameter of $0.81\,{\mathrm{nm}} \, > \, {d} \ge 0.95\,{\mathrm{nm}}$ is determined for the hard-sphere model. **b** Structure of possible domains based on the assumption that the molecules (i) adopt at identical adsorption sites and (ii) experience hard-sphere repulsion. The quantity *ρ* states the molecule density within the domain. **c** AFM image with high molecule coverage of ${{\Theta }} = 0.65\,{\mathrm{nm}}^{ - 2}$. The molecules arrange in locally ordered domains. All unit cells envisioned in **b** can be identified in the image.

In the following, we will examine how well such a simple hard-sphere repulsion model coincides with our observation of locally ordered domains. First, we determine the diameter of the hard sphere, which is supposed to represent an adsorbed MoMo molecule in our model. Figure 5a indicates the periodicity of the calcite (10.4) surface. Each crossing point of the gray grid designates a possible molecule adsorption position, i.e., a possible center of an adsorbed molecule. A MoMo molecule adsorbed on the surface (molecule center marked by a red dot in Fig. 5a) will block some of the neighboring adsorption positions for other molecules. In our measurements, we found that the closest nonblocked adsorption site is 0.95 nm away. Consequently, at this distance repulsive interactions must have almost decayed. Hence, this distance gives an upper bound for the molecule diameter. To also provide a lower bound, we need to identify the most distant blocked adsorption position. Therefore, we refer to Fig. 5a and notice that the most distant blocked adsorption position is 0.81 nm away from the molecule center. Hence, for our hard-sphere model, the diameter is in the range of by $0.81\,{\mathrm{nm}} \, < \, d \le 0.95\,{\mathrm{nm}}$.

This experimentally determined diameter agrees well with the DFT calculations of two MoMo molecules at varying distances, i.e., when moving away from each other along the $\left[ {42\bar 1} \right]$ direction (see Supplementary Discussion, DFT Calculations of Intermolecular Interaction, Supplementary Fig. 13): The calculations show that an intermolecular repulsion vanishes at center to center distances larger than about $d_{\mathrm{DFT}} = 0.91$ nm. The intermolecular repulsion at smaller distances originates from an electrostatic interaction between two methyl groups of the interacting molecules and continuously decreases with increasing molecule–molecule distance. Based on these DFT calculations, we can recognize that the molecules slightly differ from ideal hard spheres. In fact, the molecule density can be increased to some extent under pressure, because the short-range repulsion increases continuously and not abruptly when two molecules are brought together (see Supplementary Fig. 13). Therefore, MoMo molecules might be rather described as slightly compressible rubber balls instead of rigid spheres. However, the lack of any noticeable intermolecular attraction and the determined short-range repulsion of two MoMo molecules still strongly motivates to use a hard-sphere approximation.

Such a hard-sphere approach results in various periodic structures. In Fig. 5b, seven densely packed unit cells, labeled Domains 1–7, are illustrated. Four of these domains exist in an identical, mirror imaged configuration, labeled with an asterisk. Domains 1, 2, 4, and 7 all have an equal molecule coverage of ${{\Theta }} = 0.82\,{\mathrm{nm}}^{ - 2}$, while Domain 5 is less densely packed (${{\Theta }} = 0.71\,{\mathrm{nm}}^{ - 2}$) and Domain 6 is more densely packed (${{\Theta }} = 0.99\,{\mathrm{nm}}^{ - 2}$). The most densely packed structure is Domain 3 with ${{\Theta }} = 1.23\,{\mathrm{nm}}^{ - 2}$. In principle, other less densely packed unit cells are also possible. However, at low coverage, a hard-sphere interaction does not lead to a noticeable order.

If the assumptions of a single adsorption position and hard-sphere repulsion are correct, we should be able to find some or all of the above-mentioned domains in the locally ordered areas in our images. To inspect whether this simple picture might be useful to explain the experimental findings, we analyze an AFM image taken at high molecular coverage and close tip–sample distance in order to benefit from contrast inversion for imaging the precise molecule position and orientation. As shown in Fig. 5c, the molecule coverage is very high (0.65 molecules per nm^2^), resulting in many areas exhibiting local order. Similar to Fig. 3a, due to nonlinear thermal drift the molecule positions are slightly off compared to the expected favored adsorption positions (see Supplementary Discussion, Non-linear Thermal Drift for more details). When marking the center position of the molecules, we can, indeed, identify the above envisioned densely packed configurations on the surface. This finding corroborates our interpretation of hard-sphere repulsion. In the absence of intermolecular attraction, the potential energy does not depend on the specific molecular arrangement on the surface, given that the molecules always occupy the same adsorption position. Therefore, the here observed ordered arrangements at high coverage are caused by the confinement of hard spheres rather than by attractive interaction between the molecules^37^. Local dense packing of some of the spheres increases the number of possibilities to arrange the others. Hence, configurations with some molecules densely packed contribute with a larger number of microstates compared to configurations, where all molecules are more or less evenly spaced^37^. Therefore, configurations with ordered domains represent the typical microstate of confined hard spheres as observed in our experiment (Fig. 5c) as well as in simulation as will be shown in the following.

For a further comparison between experiment and our model, we have performed a Metropolis–Monte–Carlo simulation of ideal hard spheres. An interactive simulation can be found online (10.4119/unibi/2945083) (see also Supplementary Discussion, Hard-Sphere Simulation for details about the hard-sphere simulation). Comparing simulations with experimental results at the same coverage reveals similar structures. At high coverage (see Fig. 5c), the same locally ordered domains are observed in both cases. However, in the simulation for ideal hard-sphere repulsion, the most dense domain, Domain 3, is observed more often compared to the domains with lower density. In contrast to the simulation, in Fig. 5c, large areas are covered by low-density domains, e.g., Domain 4 and Domain 6, and Domain 3 is comparably rare. This deviation between experiment and model can be readily explained by the slight deviation of the MoMo molecules from the idealized model of incompressible spheres.

## Discussion

In this work, we provide experimental and theoretical evidence for anchoring individual MoMo molecules onto the (10.4) surface of the bulk insulator calcite held at room temperature. Our AFM results indicate that the molecules neither diffuse nor rotate at room temperature, illustrating the strong molecule–surface interaction. High-resolution images taken at close tip–sample distances reveal a cross-like inner structure of the molecules. From these high-resolution images, we deduce that the molecules lie flat on the surface and adopt an identical adsorption position and orientation. The strong molecule–substrate interaction and the observed orientation can be readily explained by a simple electrostatic picture, illustrating the very close match between the charge distribution within the molecule and on the surface. Annealing experiments show that the molecules do not form islands even if they have enough energy to overcome the diffusion barrier. This finding can be explained by the absence of a notable intermolecular attraction. In fact, when assuming identical adsorption positions and a hard-sphere repulsion between the molecules, ordered molecular domains can be envisioned, which we indeed find in high-coverage structures. Our results, thus, demonstrate that the formation of ordered arrays of this metal-complexing molecule is driven by confinement, rather than by intermolecular attraction. This study contributes to exploring alternative strategies for creating ordered metal arrays on electrically insulating support surfaces.

## Methods

### Experimental section

All AFM measurements shown in this work were carried out with a Scienta Omicron VT AFM XA operated in the frequency modulation mode^38^. Sample preparation as well as molecule deposition were performed in situ with a chamber base pressure typically better than 10^−10^ mbar. All temperatures stated in this work were measured with a Pt-100 sensor located at the sample holder stage with an accuracy of ±1 K. Note that the temperature at the sample surface will differ from this read-out. Optical quality calcite (CaCO_3_) crystals were purchased from Korth Kristalle GmbH, Kiel, Germany. After insertion into the UHV chamber, the crystals were annealed at 700 K to remove air-borne contaminants before in situ cleavage. Prior to molecule deposition, the crystal was annealed at 650 K for 1 h. The dimolybdenum tetraacetate (MoMo) molecules were bought from Aldrich, Munich, Germany, and were used after thorough degassing. Molecule deposition was done using a home-built Knudsen cell that is heated to ~440 K for molecule sublimation. The coverages shown in this work were achieved by sublimation times ranging from 10 to 50 min with the cell being positioned ~9 cm away from the sample.

### Computational methods

DFT calculations were performed with the planewave-pseudopotential package Quantum ESPRESSO^39^, using Ultrasoft pseudopotentials^40^ with a wave function (charge) kinetic energy cutoff of 408 eV (4080 eV) and a GGA–PBE^41^ exchange–correlation functional. The Grimme-D2 van der Waals interaction^42^ was included. The Brillouin zone was sampled with the **k** = Γ point. Calcite (10.4) was modeled with a periodically repeated slab of three layers, with a vacuum gap between the adsorbed molecule and the bottom layer of the slab replica of ~15 Å. Only forces on molecule atoms and surface atoms belonging to the first two layers were allowed to relax, up to 0.026 eV/Å. A smearing of 0.16 eV was used to improve convergence in the electronic iterations. The NEB energy barriers have been calculated using five images/replicas^43^.

## Supplementary information

Supplementary Information

## Data Availability

The data that support the findings of this study are available from the corresponding author upon reasonable request.
