# Supplementary material for: Creating a regular array of metal-complexing molecules on an insulator surface at room temperature
Source: Nat Commun. 2020 Dec 21;11:6424. doi: 10.1038/s41467-020-20189-x (PMC7752910; doi:10.1038/s41467-020-20189-x)
Supplement: Supplementary file 1 — Supplementary Information [file 41467_2020_20189_MOESM1_ESM.pdf]

SUPPLEMENTARY INFORMATION

Creating a regular array of metal-complexing molecules on an insulator surface at room temperature

Simon Aeschlimann,[1,2] Sebastian V. Bauer,[1] Maximilian Vogtland,[3] Benjamin Stadtmüller,[4] Martin Aeschlimann,[4] Andrea Floris,[5] Ralf Bechstein,[3] Angelika Kühnle[3]*

[1] Institute of Physical Chemistry, Johannes Gutenberg University Mainz, Duesbergweg 10-14, 55099 Mainz, Germany

[2] Graduate School Materials Science in Mainz, Staudingerweg 9, 55128 Mainz, Germany

[3] Physical Chemistry I, Department of Chemistry, Bielefeld University, Universitätsstraße 25, 33615 Bielefeld

[4] Department of Physics and Research Center OPTIMAS, University of Kaiserslautern, Erwin-Schrödinger-Straße 46, 67663 Kaiserslautern, Germany

[5] School of Chemistry, University of Lincoln, Brayford Pool, Lincoln LN67 PS, UK

*Corresponding author: angelika.kuehnle@uni-bielefeld.de

Supplementary Discussion

I. DFT Calculations of Single MoMo on Calcite (10.4)

DFT atomic relaxations were performed to identify the most stable adsorption geometry of a single MoMo molecule on calcite (10.4). The molecule was relaxed on the surface starting from 64 non-equivalent initial configurations (see Supplementary Figure 1). The energy of the most stable configuration (shown in Figure 3d of the main text) is set to $E = 0$ eV. The first group of geometries corresponds to $E < 0.2$ eV. Note that some different initial configurations relax in the same final geometry. For instance, 40, 41, 56 (E= 0 eV) or 16, 48 (E= 30 meV) or 14, 62 (E= 10 meV). These configurations have the Mo atom on top of a carbonate group and four MoMo oxygens bind with the neighbouring Ca atoms. Some configurations are very close in energy. This stems from two facts: *i)* the molecule is slightly tilted as it aligns to one of the two diagonals connecting two Ca atoms (this gives rise to two slightly different orientations and energies); *ii)* the molecule can adsorb in two non-equivalent $[0\bar{1}0]$ rows (mirrored rows). At higher energies ($0.2 < E < 0.5$ eV), the molecule is not on top of the carbonate group (see Supplementary Figures 4-6) but keeps a similar orientation as for more stable geometries. At even larger energies ($E > 0.5$ eV), the molecule can be rotated by 45° (with respect to its stable geometry, with only two MoMo oxygens bound with the Ca atoms) or can be in bridge position between two Ca atoms.

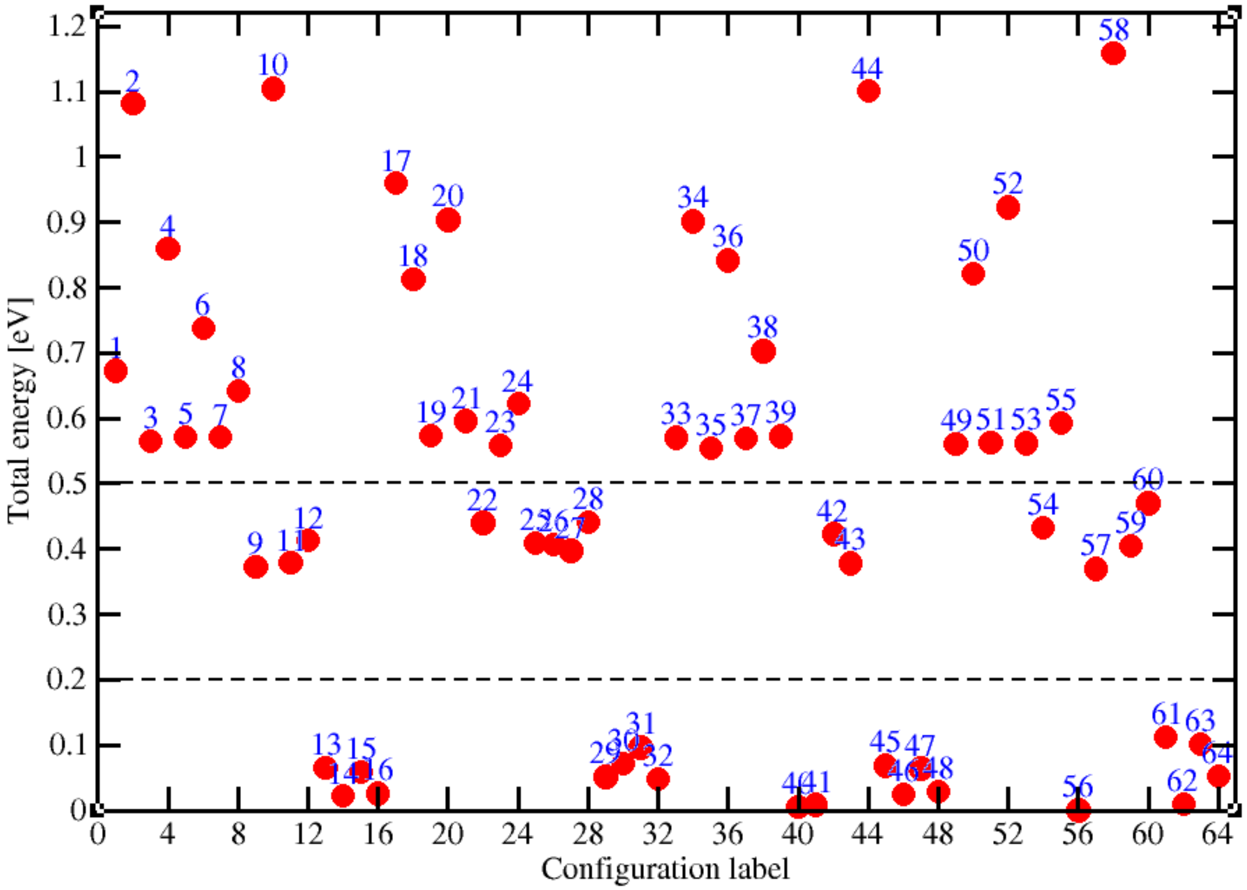

Supplementary Figure 1. DFT total energies of optimized geometries of a single MoMo molecule on calcite (10.4) calculated from 64 non-equivalent initial configurations. Configurations are labelled from 1 to 64. The energies are referred to the most stable configurations (40, 41, 56), where we set *E*=0 eV.

Stable configurations of MoMo on calcite (10.4)

In the following we present several examples of stable and less stable configurations of MoMo on calcite (10.4). The configuration labelling refers to the one used in Supplementary Figure 1.

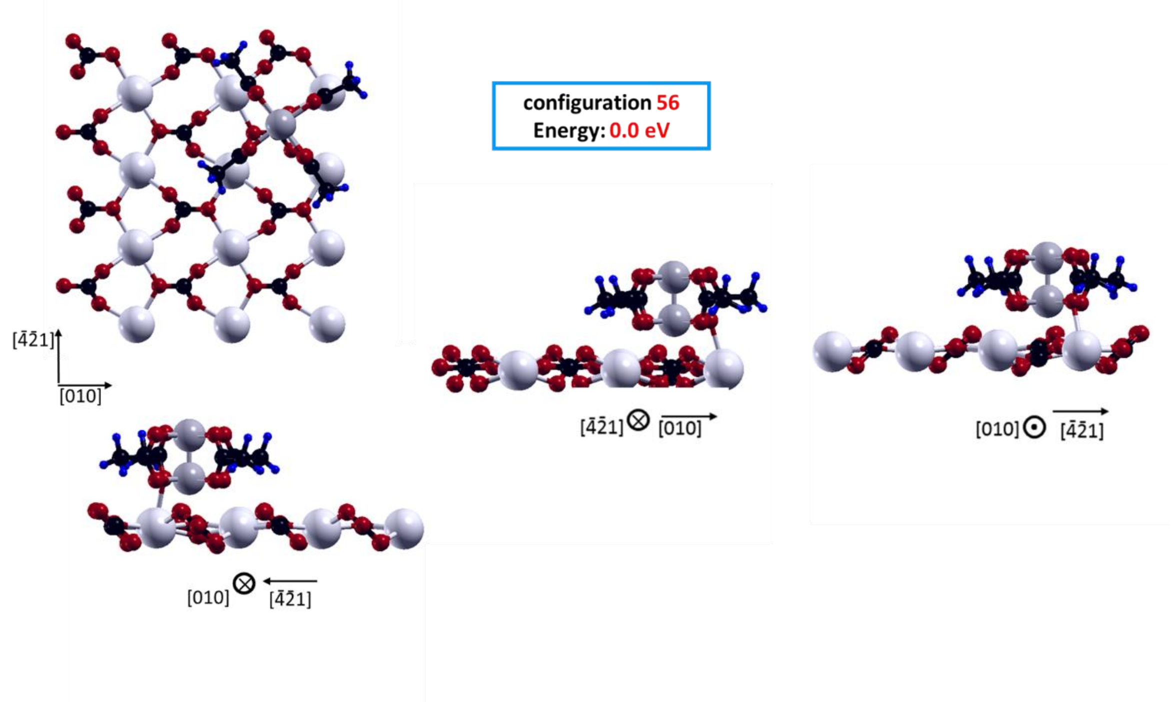

Supplementary Figure 2. Most stable configuration of MoMo on calcite (10.4) (equivalent to configuration in Figure 3d of the main text, here the Mo atoms are in grey).

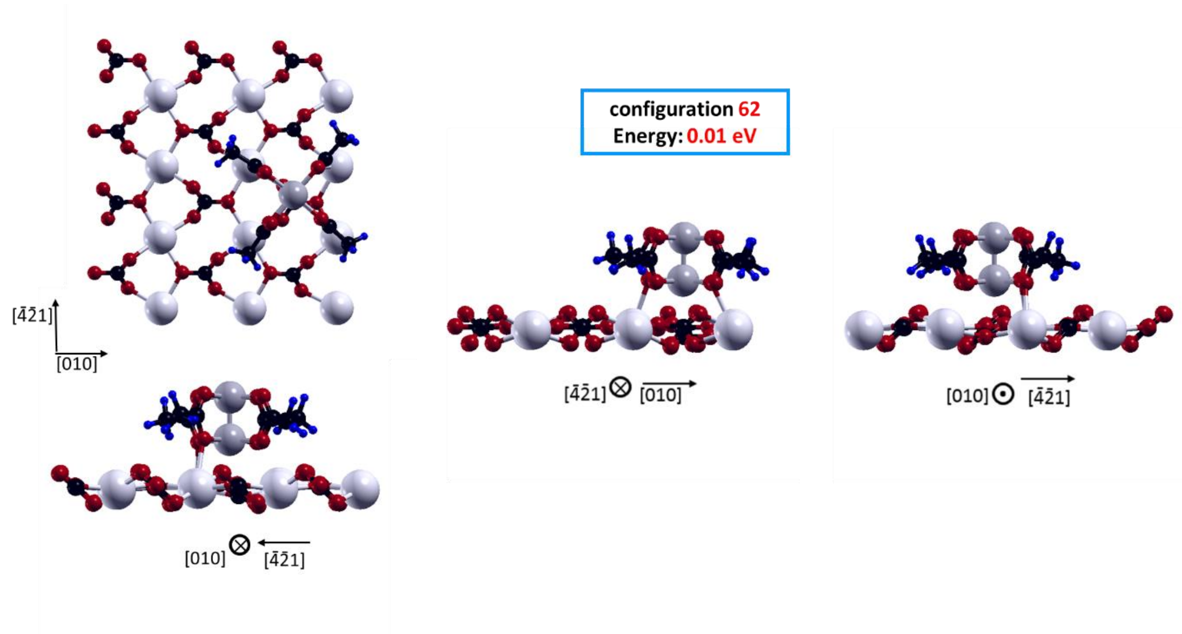

Supplementary Figure 3. A stable configuration of MoMo on calcite (10.4) (similar to the one in Figure 3d of the main text, but adsorbed in a non-equivalent, mirrored row).

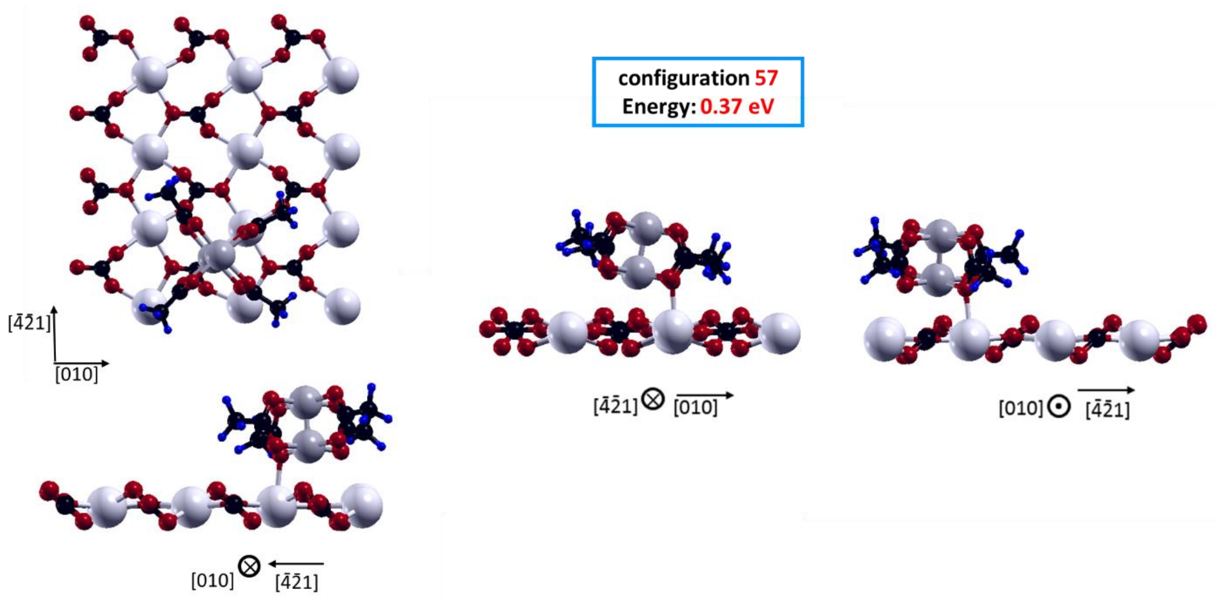

Supplementary Figure 4. A configuration of MoMo on calcite (10.4) representative of a higher energy interval (0.2 < *E* < 0.5 eV). The molecule is not on top of the carbonate group but keeps a similar orientation as for more stable geometries.

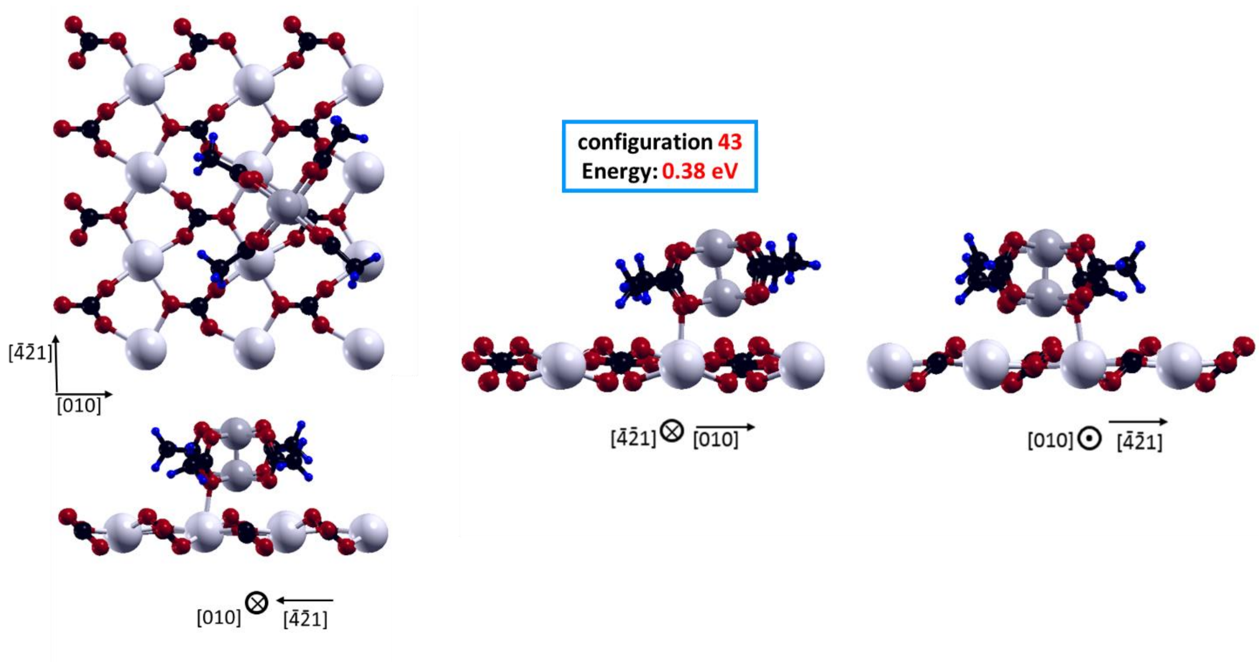

Supplementary Figure 5. Another configuration of MoMo on calcite (10.4) representative of a high energy interval (0.2 < *E* < 0.5 eV). The molecule is not on top of the carbonate group but keeps a similar orientation as for more stable geometries.

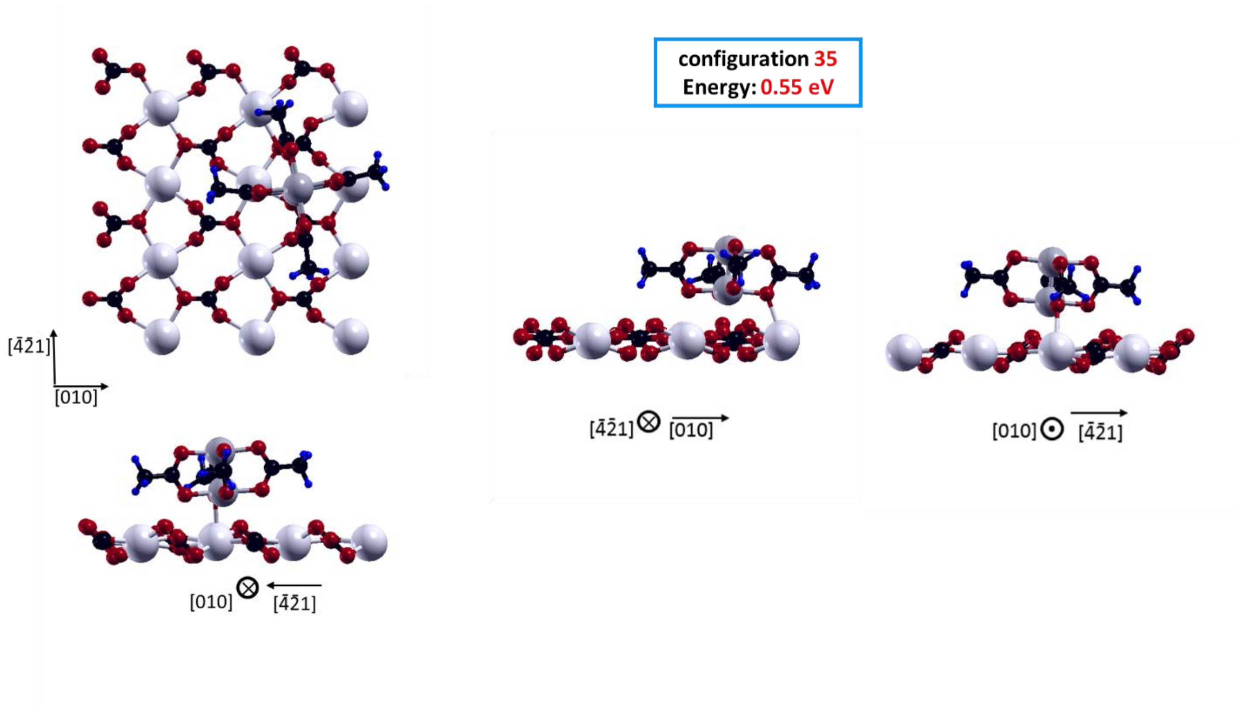

Supplementary Figure 6. configuration of MoMo on calcite (10.4) representative of even higher energy interval (*E* > 0.5 eV). The molecule is rotated by 45° with respect to the most stable geometry, with only two MoMo oxygens bound with the Ca atoms.

II. Löwdin Charge Analysis, Charge Density and Charge Displacement Field

We calculated the atomic Löwdin charges of the entire system (molecule adsorbed on the surface), which we compare with the number of valence electrons of the corresponding elements (see Supplementary Table 1 and Supplementary Figure 7).

These charges indicate a dominant electrostatic component in the molecule-surface (M-S) interaction (see also below). The electrostatics, in turn, can be decomposed in several contributions, enhanced due to the excellent M-S structural matching: The positive ($\approx +1.0$ e) lower Mo atom sits on top of an overall negative carbonate group (≈ -0.8 e); the negative oxygen atoms belonging to the acetate group (≈ -0.4 e) interact with the positive Ca atoms

($\approx +1.0$ e); the positive H atoms ($\approx +0.2$ e) in the MoMo methyl groups interact with the negative carbonate groups (≈ -0.8 e).

Element	Number of valence electrons Z_{val} (e)	Löwdin charge (e)	Z_{val} – Löwdin (e)
Ca	10	8.98/9.04	1.02/0.96
C_{calcite}	4	3.41/3.44	0.59/0.56
O_{calcite}	6	6.43/6.48	-0.43/-0.48
Mo	14	13.03/13.13	0.97/0.87
$C_{\text{MoMo -CH3}}$	4	4.44	-0.44
$C_{\text{MoMo -CO2}}$	4	3.52	0.48
O_{MoMo}	6	6.37/6.39	-0.37/-0.39
H	1	0.79/0.8	0.21/0.2

Supplementary Table 1. Number of valence electrons, calculated Löwdin charges and their differences (partial charges).

In order to corroborate the fact that the dominant M-S interaction is of electrostatic nature, in Supplementary Figure 8 we present the charge density of the system at three different isosurface values. From all three plots we can see that the density profiles follow the atomic profiles, even at a low-density value (0.013 a_0^{-3}, where a_0 denotes the Bohr radius) with respect to the density maximum value (1.04 a_0^{-3}). This lack of charge between the substrate and the molecule suggests again the absence of a covalent component between the two. From the 0.5 a_0^{-3} high density isosurface (Supplementary Figure 3, left) the atoms which contain more charge, as expected, are Mo, the oxygens close to it and, on the surface, the Ca and O atoms.

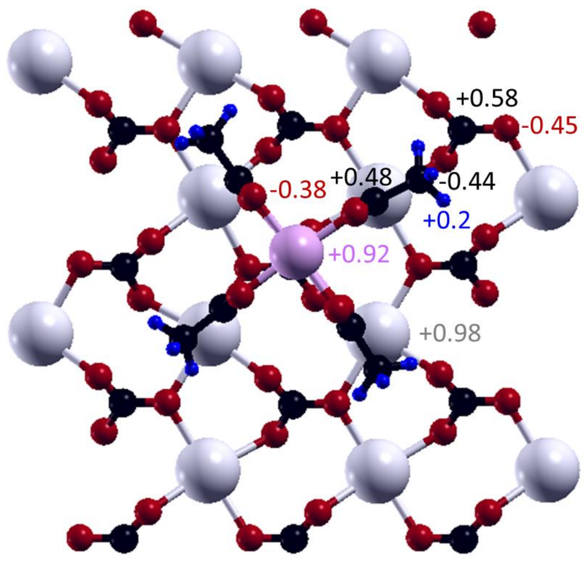

Supplementary Figure 7. Difference between calculated Löwdin charges and nominal valence of the atoms in the system (partial charges). The charges color code matches the one of the corresponding atoms.

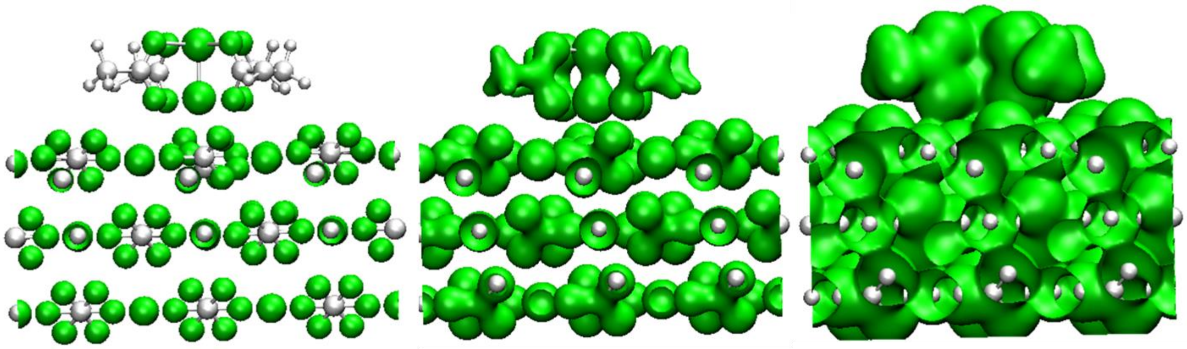

Supplementary Figure 8. Charge density isosurfaces (green) at 0.5 a_0^{-3}(left panel), 0.13 a_0^{-3} (middle panel) and 0.013 a_0^{-3} (right panel). Maximum isosurface value: 1.04 a_0^{-3}.

Importantly, we find that Löwdin charges of the fully interacting system, are extremely similar to the charges of the calcite and MoMo when they are calculated alone in the unit cell in their adsorbed geometries: charge differences are typically of the order of 10^{-3}e and only in some cases 10^{-2}e). This indicates a low, net amount of M-S charge displacement (see also below),

thus the much larger displacements shown in Supplementary Table 1 result from an internal redistribution of charge in each subsystem, molecule and substrate.

Even if, from the Löwdin charges analysis, the charge displacement upon adsorption is minimal, it is interesting to analyze this aspect further.

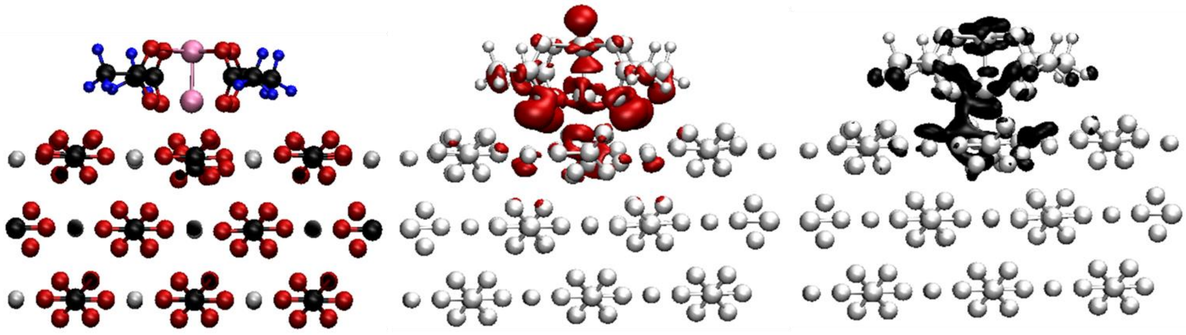

Supplementary Figure 9. Charge density displacement field upon MoMo adsorption on calcite. Left panel: adsorbed structure, as a reference. Middle panel: positive isosurface value at 0.0013 a_0^{-3} (in red) indicates charge accumulation. Right panel: negative isosurface value at -0.0013 a_0^{-3} (in black) shows charge depletion. The maximum/minimum isosurface values are $\pm 0.02\ a_0^{-3}$.

In Supplementary Figure 9, we present the charge density displacement field. The middle and right panels' plots are isosurfaces of the density difference: $\Delta\rho(r) = \rho(r)_{int} - [\rho(r)_{calcite} + \rho(r)_{MoMo}]$ where $\rho(r)_{int}$ is the electronic charge density of the fully interacting system; $\rho(r)_{calcite}$ and $\rho(r)_{MoMo}$ are the electronic densities of calcite and MoMo molecule, calculated separately and alone in the unit cell, *i.e.* non interacting (but in their adsorbed geometry). Positive $\Delta\rho(r)$ isosurface values (in red) indicate a charge accumulation upon adsorption, negative values (in black) a charge depletion. We first notice, on a qualitative basis, that the amount of displaced charge is relatively small compared to the total density of charge (Supplementary Figure 3), as the maximum/minimum values of the displaced charge isosurfaces

are ± 0.02. This again points to a lack of covalent bonding upon adsorption. However, Supplementary Figure 9 still highlights a complex pattern of charge displacement, internal to each subsystem (M and S) and between the two. The strongest feature is a charge accumulation in the O molecular atoms closer to the substrate, which become slightly more negative (Supplementary Figure 9, middle panel). A corresponding charge depletion come partly from the underlying Ca atoms and from the surface protruding O atoms, which become slightly more positive (Supplementary Figure 9, left panel). This redistribution creates a small induced downward dipole between molecule and substrate. This must reinforce the electrostatic interaction discussed above. There is also a redistribution of charge around the Mo atoms. Vertical lobes above/below the higher Mo atom are present, compensated by some other Mo orbitals that get emptier. The lower Mo atom is on top of a protruding O of the surface, which acquires charge. The Ca atoms under the molecule slightly polarize, with a more positive part towards the molecule. Overall, this complex and minute redistribution of charge upon adsorption implies a slightly enhanced electrostatic charge polarization at the M-S interface.

Importantly, hydrogens are not so much affected by a charge redistribution, their positive charge ($\approx +0.2$e) is hardly affected by the adsorption (besides a slight polarization). This goes in the direction of an unchanged, if not slightly enhanced, electrostatic M-M interaction when the molecules are adsorbed on the surface, as compared to the one that two molecules experience in the absence of the surface.

A final, important aspect is the total lack of surface charge redistribution in surface areas already some small distance away from the molecule (see Supplementary Figure 9). This clearly indicates the lack of an electronic screening, potentially affecting the M-M interaction in the presence of the surface.

III. Diffusion Energy Barriers Along the $[0\bar{1}0]$ and $[42\bar{1}]$ Directions Calculated by Nudged Elastic Band (NEB).

We calculated the diffusion energy barriers and the corresponding minimum diffusion paths of a MoMo molecule along the crystallographic directions $[0\bar{1}0]$ and $[42\bar{1}]$ of calcite (10.4) (see Supplementary Figures 10 and 11, respectively). The energy barrier separating two equivalent adsorption sites along $[0\bar{1}0]$ is 1.17 eV. The initial and final states correspond to the configuration in Figure 3d of the main text. The barrier separating two non-equivalent (but degenerate due to the glide-reflection symmetry) adsorption sites being half a unit cell apart along $[42\bar{1}]$ is 0.88 eV. The difference in the barrier values along these directions is related to the electrostatic repulsion between the positively charged MoMo molecule center and the positively charged calcium ions of the substrate. The smaller the distance between the molecule's diffusion path to the calcium ions, the higher the barrier. For diffusion along $[0\bar{1}0]$ and $[42\bar{1}]$ directions the molecule must pass between two Ca atoms being separated by 0.4 nm and 0.5 nm, respectively. Hence, the diffusion barrier is higher along the $[0\bar{1}0]$ direction.

The diffusion barriers determined by NEB calculations are in excellent agreement with the experiment. In the AFM image series, an anisotropic molecule diffusion along the $[42\bar{1}]$ direction is observed, with a corresponding smallest diffusion barrier of 1.0 eV. At room temperature, no noticeable molecule movement along the $[0\bar{1}0]$ direction is found, indicating a comparatively larger diffusion barrier along this direction.

Barriers along different (*e.g.* diagonal) directions are expected to be much higher, as the molecule consisting of a partially positive charged molybdenum center would have to move on top of another partially positive charged Ca atom.

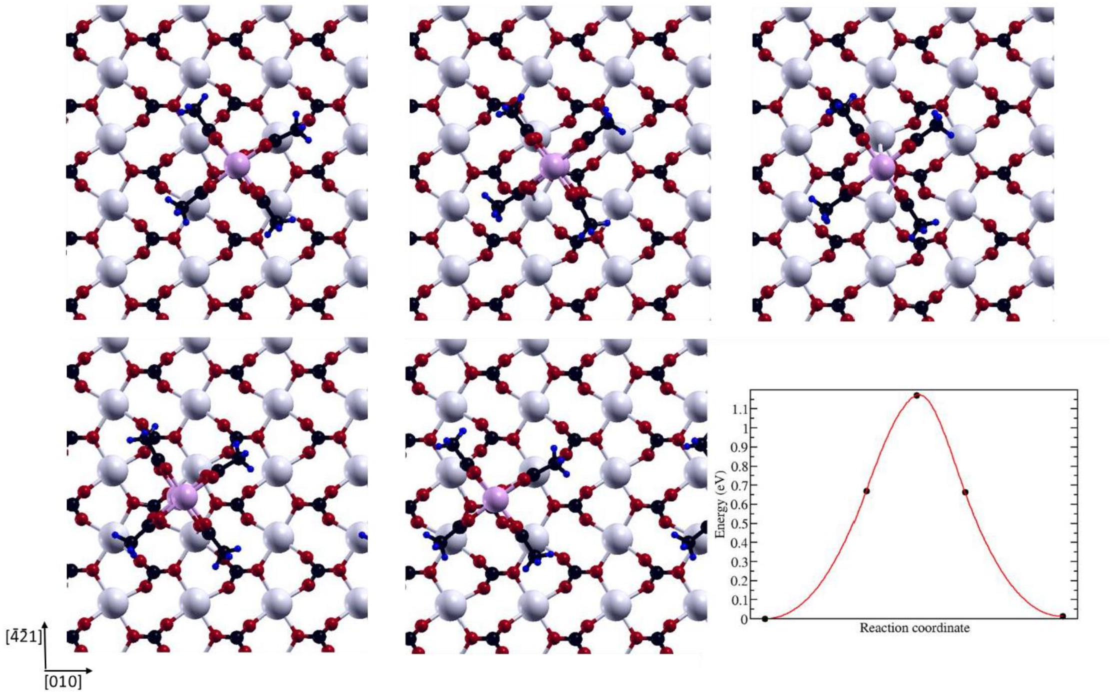

Supplementary Figure 10. Minimum diffusion paths of a MoMo molecule diffusing along the $[0\bar{1}0]$ direction of calcite (10.4), between the two stable equivalent configurations (panels from top left to bottom middle). The molecule moves from right to left. The bottom-right panel shows the corresponding minimum energy profile, with a calculated barrier of 1.17 eV. The red plot is an interpolation of the five images/replicas used in the NEB calculation.

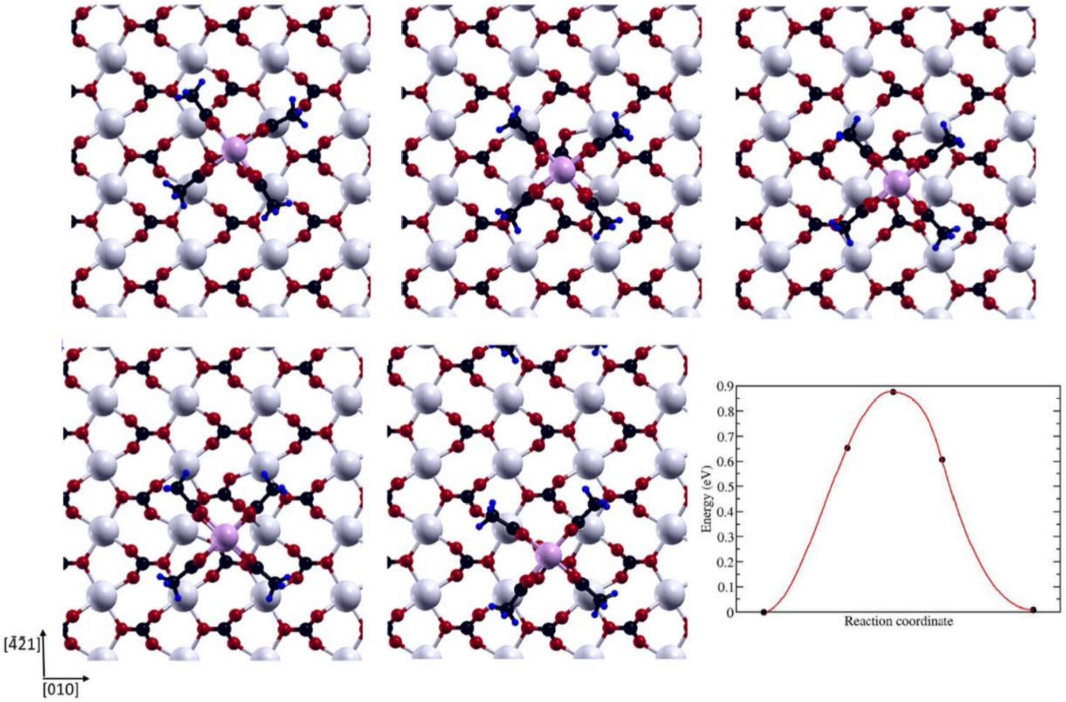

Supplementary Figure 11. Minimum diffusion paths of a MoMo molecule diffusing along the $[42\bar{1}]$ direction of calcite (10.4), between two stable configurations (panels top left to bottom middle) half unit cell apart along $[42\bar{1}]$. Configurations in panels top left and bottom middle are inequivalent but degenerate within the DFT numerical error ($\Delta E < 0.01$ eV/cell). The molecule moves from top to bottom. The bottom-right panel shows the corresponding minimum energy profile, with a calculated barrier of 0.88 eV. The red plot is an interpolation of the five images/replicas used in the NEB calculation.

IV. Diffusion Analysis at 327 K

As shown in this work in Figure 4 of the main text, at room temperature about 3 % of the molecules have changed position along the $[42\bar{1}]$ direction between 16 min. This corresponds to a diffusion rate of about $\nu_{\mathrm{diff,\,300K}} = 3.5 \cdot 10^{-5}\ \mathrm{s}^{-1}$. In addition to the diffusion analysis at 300 K presented in the manuscript, we have performed further AFM measurements at 327 K to determine the hopping rate at elevated temperatures.

Image pair for comparison	Number of moved molecules	Total number of molecules	Percentage of moved molecules
1 and 2	45	152	30 %
2 and 3	56	178	31 %
3 and 4	62	192	32 %
4 and 5	59	170	34 %
$\sum$	222	692	32 %

Supplementary Table 2. Percentage of molecules moving between two consecutive images with a time difference of 16 min. On average about 32 % of the molecules have moved between two images at 327 K.

By comparing five consecutives AFM images at 327 K (see Supplementary Table 2), we have obtained a percentage of 32 % of molecules, which have moved between 16 min. Consequently, about 68 % of the molecules did not change position at all, while the other molecules have jumped once, twice or even more often during this time interval. The probability $P_\lambda(k)$ of a given number of jumps k occurring in a fixed time interval can be described by a Poisson distribution

$$P_\lambda(k) = \frac{\lambda^k}{k!} e^{-\lambda} \tag{1}$$

where λ is the expected value and, hence, in our case the average number of jumps in a time interval. From the number of non-moving molecules, we can calculate the expected value of jumps λ for a time interval of 16 min:

$$P_\lambda(0) = \frac{\lambda^0}{0!} e^{-\lambda} = 0.68 \tag{2}$$

$$\lambda = 0.38$$

This results in an average hopping rate of

$$\nu_{\text{diff, 327K}} = \frac{0.38}{16 \cdot 60}\,\text{s}^{-1} = 4.0 \cdot 10^{-4}\,\text{s}^{-1} \tag{3}$$

at 327 K. At this temperature, the molecule seem to have a clearly higher mobility than at 300 K. Assuming an attempt frequency of $\nu_0 = 10^{12}\,\text{s}^{-1}$ results again in a diffusion barrier of $E_{\text{diff}} = 1.0\,\text{eV}$ and, hence is in perfect agreement with the determined diffusion barrier at room temperature.

This analysis clearly shows that the molecules have a sufficient mobility at 400 K to arrive at their ideal adsorption position during the experiment time.

V. Annealing Experiment

Annealing experiments of MoMo on calcite (10.4) are performed to investigate whether island formation can be induced upon heating. In these experiments, we anneal the sample at a given temperature for one hour, let it cool down to room temperature and image the surface. This procedure is repeated with increasing annealing temperatures to observe the resulting structural changes in the molecular pattern on the surface. As shown in Supplementary Figure 12, AFM images are obtained at room temperature subsequent to annealing to 300 K, 356 K, 492 K, 628 K, 698 K and 738 K. Basically, no change in the random molecule distribution is observed up to an annealing temperature of approximately 700 K. However, above 700 K large cluster are formed, which we tentatively ascribe to a decomposition of the molecules and a clustering of the arising fragments.

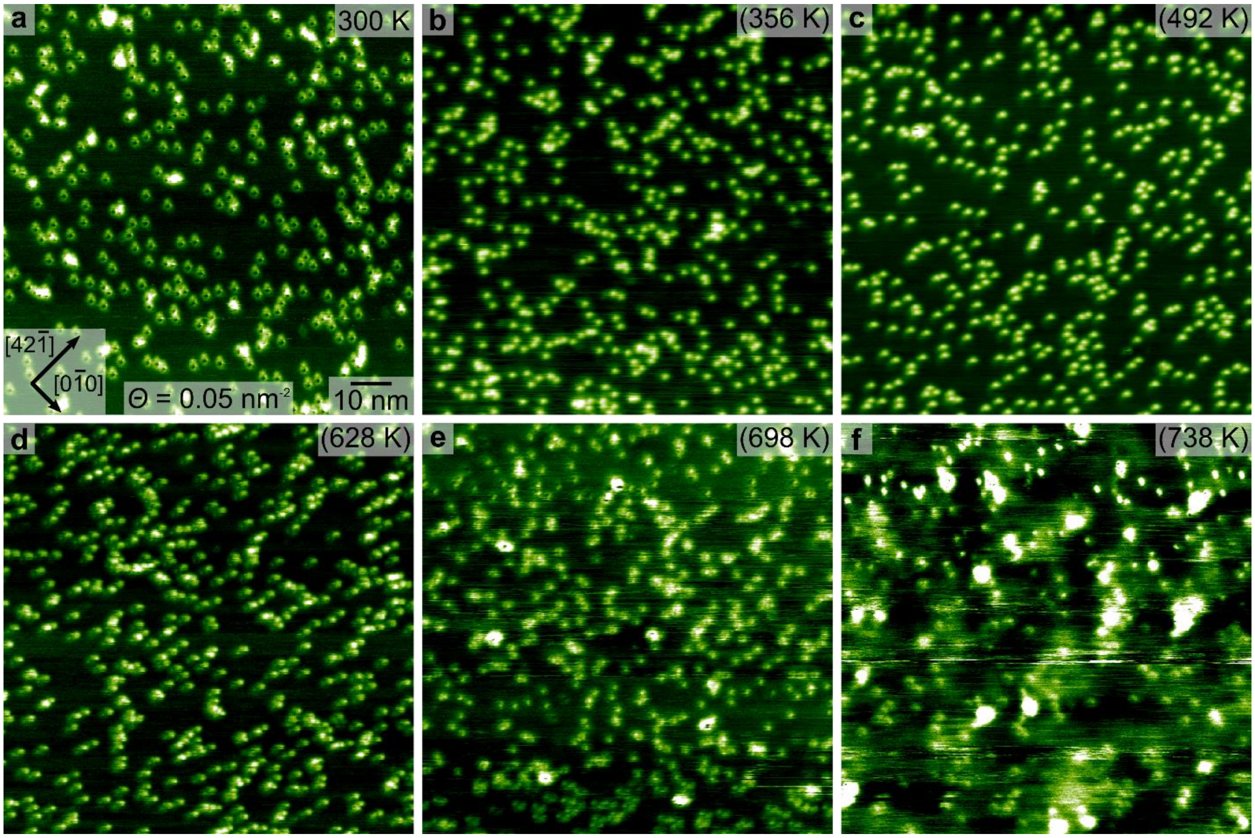

Supplementary Figure 12. AFM images of MoMo on calcite (10.4) at room temperature subsequent to annealing for one hour at a) 300 K, b) 356 K, c) 492 K, d) 628 K, e) 698 K and f) 738 K. a)-e) The qualitative molecule arrangement in a randomly scattered fashion remains basically unchanged up to a temperature of approximately 700 K. f) Above 700 K, large clusters are formed, for that reason we had difficulties to regain stable imaging conditions.

VI. DFT Calculations of Intermolecular Interaction

In this Section, we evaluate via DFT the repulsive energy between two MoMo molecules. Our aim is to ascertain the validity of the hard-sphere repulsive model illustrated in the main text and detailed in the Supplementary Discussion, VIII. Hard-Sphere Simulation. In Supplementary Figure 13 we plot the total energy of the two interacting molecules in gas phase with a fixed orientation, as a function of the distance between their centres along the $[42\bar{1}]$ direction. We

adopt the orientation of the most stable adsorption geometry found on the surface (see Figure 3d of the main text). We keep the orientation fixed (the molecule atoms can only move along $[42\bar{1}]$) to reproduce the constraints imposed by the surface. Based on the charge analysis shown in the Supplementary Discussion, II. Löwdin Charge Analysis, Charge Density and Charge Displacement Field, we can exclude further surface mediated effects like electron screening. We start from a short distance (0.81 nm, experimentally not observed) and compute the total energy until no further change in energy can be detected. The repulsion, vanishing at $d_{\mathrm{DFT}} = 0.91$ nm, is related to the electrostatic interaction between the CH_3 groups. Note that when the molecules are allowed to rotate in gas phase (not shown) they manage to relax by keeping a short distance (0.855 nm). However, on the surface this would be energetically unfavourable, as the change in orientation would make the MoMo interaction with the surface Ca atoms far less optimal. As shown in Supplementary Figure 13, the short-range repulsion increases continuously when two MoMo molecules are brought together and not as abruptly as for a rigid sphere. Therefore, in contrast to ideal hard-spheres, MoMo molecules can be slightly compressed under sufficient pressure. However, in a first approximation it remains justified to use a hard-sphere model, because a short-range repulsion only and no intermolecular attraction is recognized.

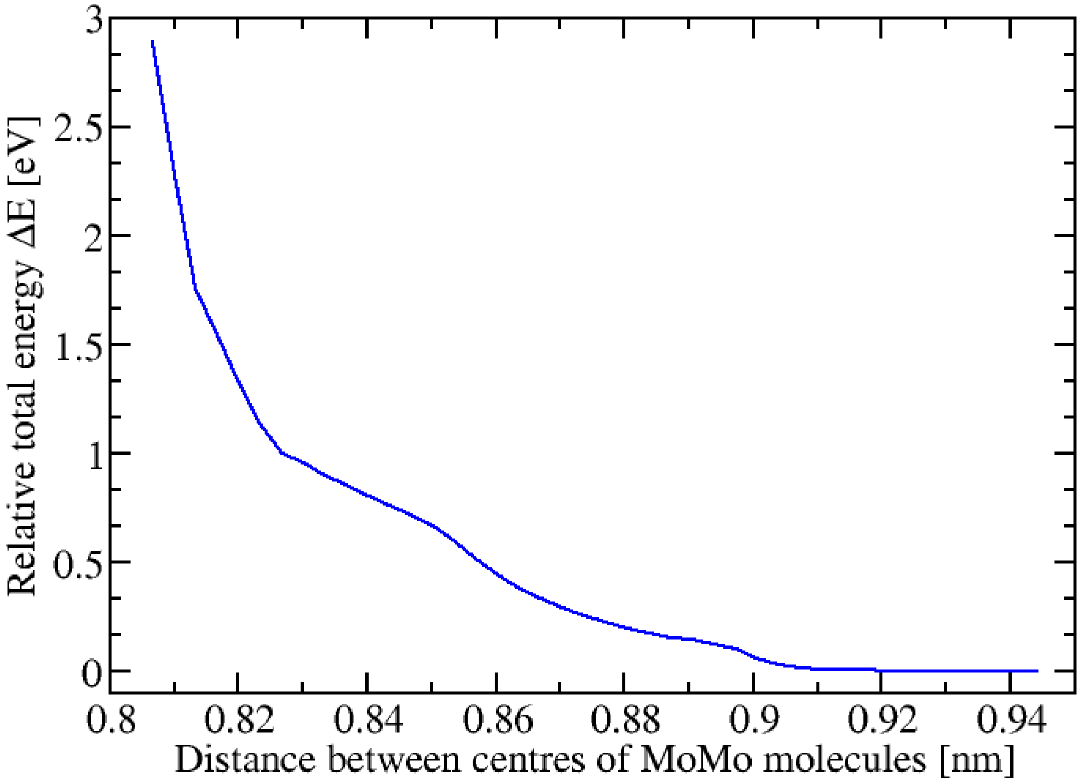

Supplementary Figure 13. Total energy of two MoMo molecules as a function of their distance *d* along the $[42\bar{1}]$ direction, calculated by DFT in gas phase. The energy is computed relative to the energy of two non-interacting molecules (for *d* > 0.91 nm), which is set to zero.

VII. Non-linear Thermal Drift

In the AFM measurements presented in this work, for example in Figure 5c of the main text, the molecule positions are slightly off compared to the expected favored adsorption positions. As we perform high-resolution AFM measurements at room temperature and as recording of a single image takes up to 8 min, we suffer from non-neglectable linear and non-linear thermal drift.

Here, we show that the apparent slight offset of the molecule adsorption positions in the AFM images is a consequence of thermal drift during the experiment and that any non-identical adsorption positions can be excluded.

It is possible to distinguish between thermal drift and non-identical adsorption positions in an AFM image by analyzing the offset of the measured adsorption positions compared to the expected ideal adsorption positions as a function of the distance to a reference molecule. For thermal drift, this offset should increase with the distance to the reference molecule and for non-identical adsorption positions the offset should be independent of the distance.

In Supplementary Figure 14, we demonstrate that the offsets in our AFM experiment are indeed caused by thermal drifts and not by the existence of different local adsorption positions. Therefore, we have marked the molecule adsorption positions as determined from the AFM image in Figure 5c of the main text, with colored crosses. Moreover, we have indicated the expected identical adsorption positions along the $[42\bar{1}]$ and the $[0\bar{1}0]$ directions by horizontal and vertical lines, respectively. Crosses adopting ideal adsorption sites with respect to the lines are marked in green, crosses having a small offset are marked in orange and crosses with a large offset are marked in red. From the two images it becomes evident that the horizontal and vertical lines are in excellent agreement with the molecule adsorption positions in the near vicinity of the two reference molecules. In contrast, an increasing offset between the lines and the molecules is visible at further distance to the left and right side. Moreover, all molecules in the same area have a similar offset. This fact further corroborates our assignment of the offset being caused by thermal drift.

From this analysis it becomes evident that the molecules indeed adopt identical molecule adsorption positions. However, the molecule positions in the AFM image are slightly displaced due to thermal drift.

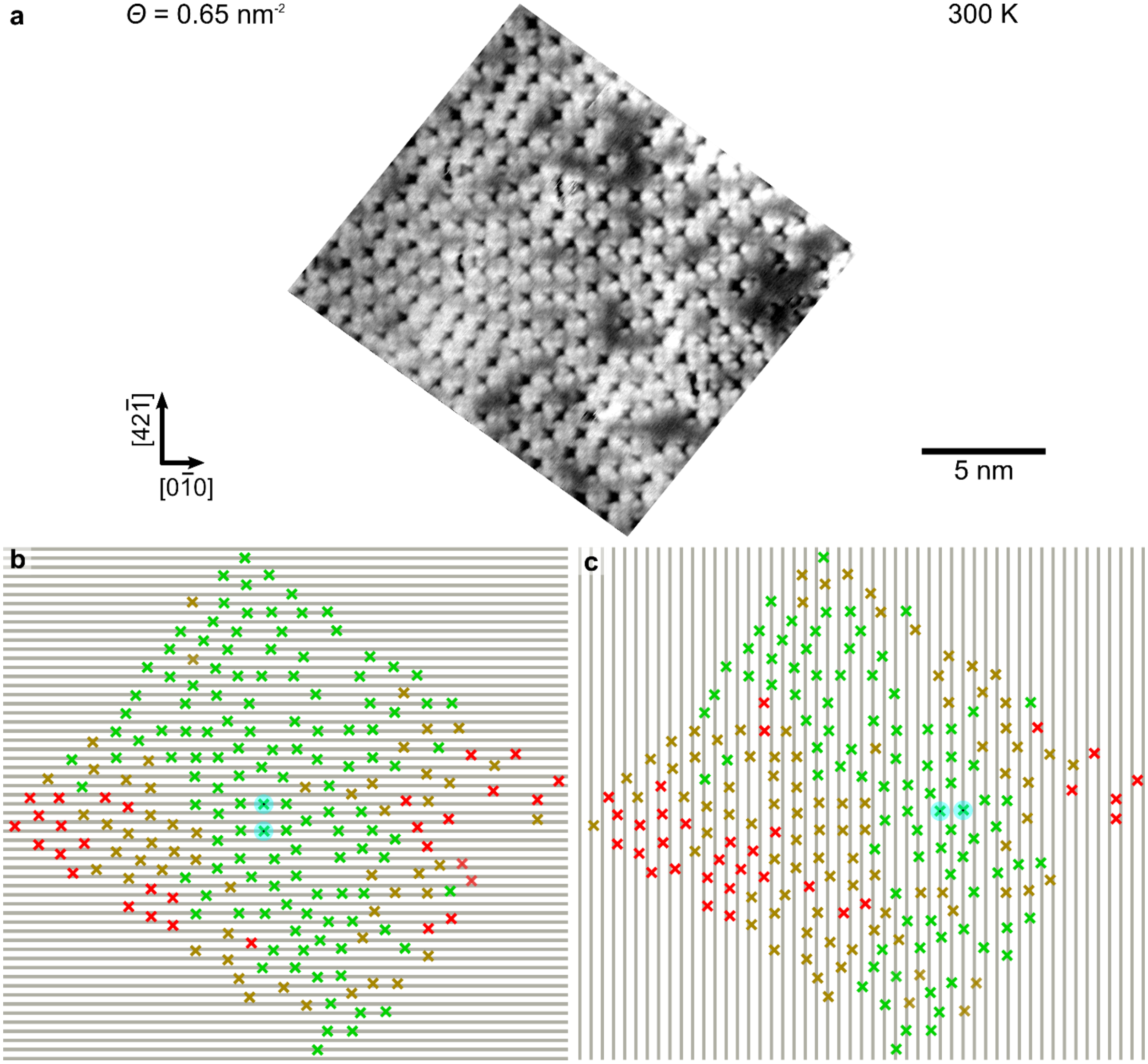

Supplementary Figure 14. Thermal drift causes a slight displacement of the measured molecule adsorption positions compared to the expected favored adsorption positions. (a) AFM image of MoMo on calcite at room temperature as shown in Fig. 5c. (b) and (c) The molecule positions in the AFM image are indicated by green (ideal position), orange (small offset) and red (large offset) crosses. The grey horizontal and vertical lines mark the favored molecule adsorptions positions along the $[42\bar{1}]$ and the $[0\bar{1}0]$ direction,

respectively. The lines are aligned with respect to the two reference molecules marked with blue circles.

VIII. Hard-Sphere Simulation

Metropolis Monte Carlo simulations of MoMo adsorbed on calcite (10.4) were performed. In these simulations, MoMo molecules were modelled as hard spheres with a diameter of $d = 0.90\ \mathrm{nm}$. According to our experimental observations, in the simulation the molecules were constrained to exclusively adsorb on specific positions on top of a carbonate group of the calcite surface. A link to the interactive simulation can be found here: “https://doi.org/10.4119/unibi/2945083”. The code is available in the accompanying JavaScript file "MoMo.js". The Supplementary Figure 15 displays three snapshots from this simulation, showing an equilibrated situation for various coverage. The crossing points of the grey grid indicate possible molecule adsorption positions. For comparison between experiment and simulation the same coverages of (a) $\Theta = 0.07\ \mathrm{nm}^{-2}$, (b) $\Theta = 0.41\ \mathrm{nm}^{-2}$ and (c) $\Theta = 0.65\ \mathrm{nm}^{-2}$ as in Figure 2 of the main text are chosen. Similar to the experiment, at low coverage (see Supplementary Figure 15a) only single molecules are obtained, exhibiting no ordered structure. Upon increasing the coverage (see Supplementary Figure 15b and c), various periodic domains are formed in the dense areas, both in the experiment as well as in the hard-sphere simulation. However, the dense domains, Domain 3 and Domain 6, occur much more frequently in the simulation than observed in the experiment. A possible explanation for this deviation

could be that the repulsion between two MoMo molecules does not decrease as isotropically and abruptly as for a perfect hard sphere.

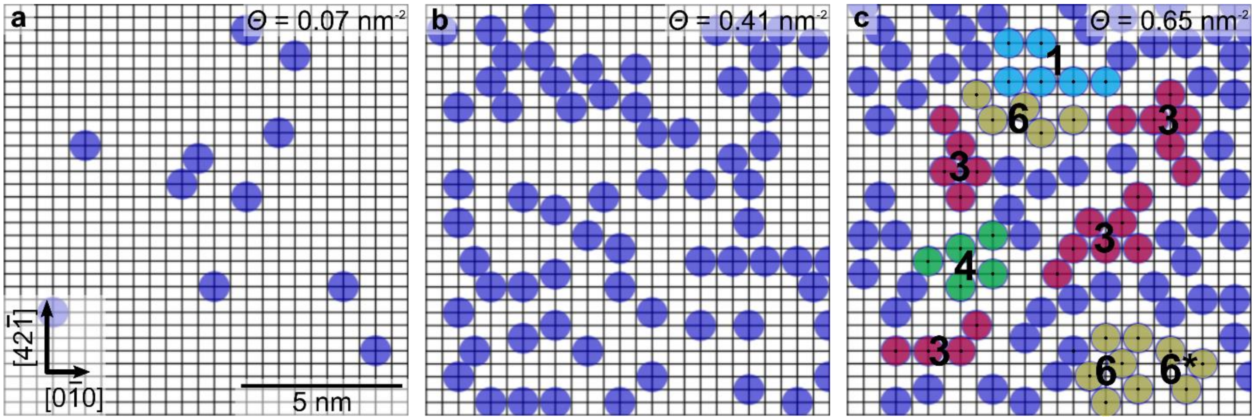

Supplementary Figure 15. Snapshots of Metropolis Monte Carlo hard-sphere simulation of MoMo adsorbed on calcite (10.4) surface at various coverage. MoMo molecules are illustrated as blue spheres with a radius of 0.90 nm. The grey grid illustrates the periodicity of the calcium ions of the calcite sample. (a) At low coverage of $\Theta = 0.07\ \mathrm{nm}^{-2}$, single molecules are obtained and no order is recognizable. (b) Upon increasing the coverage to $\Theta = 0.41\ \mathrm{nm}^{-2}$, a few denser areas, indicating first periodicity, are formed. (c) At high coverage of $\Theta = 0.65\ \mathrm{nm}^{-2}$, ordered areas are visible and marked with the corresponding domain. The most dense Domain 3 occurs most frequently.
